# *Trypanosoma vivax* in Water Buffaloes (*Bubalus bubalis*): A Host-Centered Synthesis of Pathogenesis, Epidemiology, Diagnosis, and Integrated Control with Implications for Tropical Production Systems

**DOI:** 10.3390/pathogens15030273

**Published:** 2026-03-03

**Authors:** André de Medeiros Costa Lins, Dryelle Vieira de Oliveira Brandão, Fernanda Monik Silva Martins, Aline Maia Silva, Henrique dos Anjos Bonjardim, Felipe Masiero Salvarani

**Affiliations:** 1 Instituto de Medicina Veterinária, Universidade Federal do Pará, Castanhal 68740-970, PA, Brazil; andre.lins@castanhal.ufpa.br (A.d.M.C.L.); dryelle_v.oliveira@hotmail.com (D.V.d.O.B.); nanda.monik08@gmail.com (F.M.S.M.); 2 Faculdade de Medicina Veterinária, Universidade Estadual do Ceará, Tauá 60.000.000, CE, Brazil; alinemaia.silva@uece.br; 3 Faculdade de Medicina Veterinária, Universidade do Norte do Tocantins, Araguaína 77826-612, TO, Brazil; henrique.bomjardim@ufnt.edu.br

**Keywords:** host–parasite interaction, reservoir competence, mechanical transmission, molecular diagnosis, trypanocide resistance, integrated disease management, Amazon biome model system, One Health

## Abstract

*Trypanosoma vivax* is a hemoprotozoan parasite of major veterinary importance affecting domestic ungulates in Africa and the Americas. While traditionally addressed within cattle-centered paradigms, accumulating evidence indicates that water buffaloes (*Bubalus bubalis*) are both clinically susceptible and epidemiologically significant hosts. This structured narrative review provides a host-centered synthesis of global evidence on *T. vivax* infection in buffaloes, integrating pathogenesis, transmission biology, epidemiology, diagnostics, chemotherapy, and integrated control. The analysis encompasses literature from 2000 to 2025 and incorporates seminal experimental studies published prior to 2000 that established buffalo susceptibility and reservoir competence. Evidence from cyclical (tsetse-mediated) and mechanical transmission systems is comparatively interpreted to clarify host–parasite dynamics. The Amazon biome is discussed as a model system for high-density buffalo production under mechanical vector pressure, offering case-based contextualization without geographic restriction. Particular attention is given to immunopathological mechanisms, chronic low-parasitemia carriage, diagnostic sensitivity in subclinical infections, emerging trypanocide resistance, and ecological constraints on vector control. Controversies and buffalo-specific knowledge gaps are highlighted throughout. By adopting a buffalo-centered analytical framework, this review supports translational diagnostics, targeted surveillance, and sustainable control strategies for trypanosomiasis in tropical livestock systems.

## 1. Introduction

Trypanosomiasis caused by *Trypanosoma vivax* is a major constraint to livestock production in tropical and subtropical regions of Africa and Latin America [1,2,3,4,5,6]. The parasite is transmitted either cyclically by tsetse flies (*Glossina* spp.) in endemic African regions or mechanically by hematophagous flies such as *Tabanus* spp. and *Stomoxys calcitrans*, as well as through iatrogenic practices in tsetse-free areas [3,4,7]. Clinical heterogeneity reflects host immune modulation and parasite strain diversity, fever, anemia, weight loss, reduced fertility, and decreased milk and meat productivity, generating substantial economic losses and compromising the sustainability of livestock systems in affected regions [3,4].

Although cattle are historically considered the principal domestic hosts of *T. vivax*, increasing evidence demonstrates that water buffaloes (*Bubalus bubalis*) are highly susceptible and may develop acute, subacute, and chronic infections with significant clinical and productive consequences [5,8]. In several tropical production systems, buffaloes play a central economic and socio-cultural role, particularly in regions where they represent the dominant livestock species. In the Brazilian Amazon, buffalo farming constitutes a major livestock activity and is deeply integrated into local food security and rural livelihoods [9,10,11]. In such systems, outbreaks of acute trypanosomiasis have been associated with elevated morbidity and mortality, whereas chronic and subclinical infections contribute to the maintenance of parasite circulation within and between herds [7,12]. This dual epidemiological role as both productive animals and potential long-term reservoirs highlights the necessity of a species-specific understanding of *T. vivax* infection dynamics in buffaloes.

Despite the growing recognition of buffalo involvement in *T. vivax* epidemiology, the available literature remains fragmented and predominantly cattle-centered. Most reviews addressing animal trypanosomiasis focus on bovine systems in Africa, with limited synthesis dedicated specifically to water buffaloes as a distinct host species [2,13]. Differences in management systems, ecological exposure, immune responses, and production objectives justify a host-centered analytical framework. Accordingly, this review adopts a species-focused perspective centered on *Bubalus bubalis*, integrating global evidence to contextualize infection dynamics while recognizing that epidemiological processes are inherently multi-host and regionally variable.

Within this framework, data from Africa and South America are critically synthesized to examine host–parasite interactions, transmission patterns, and control strategies relevant to buffalo production systems. The Amazon biome is not presented as a geographical restriction of this review but rather utilized as a case-based contextualization and model system of high-density buffalo production under mechanical transmission pressure. In such ecological settings characterized by floodplain environments, intense vector exposure, and mixed-species grazing transmission dynamics illustrate practical challenges in surveillance and control [4,5]. This approach allows the discussion of applied epidemiological scenarios without limiting the scope of analysis to a specific territorial fraction.

Pathogenesis in buffaloes involves complex host–parasite interactions. Antigenic variation mediated by variant surface glycoproteins (VSGs) enables immune evasion and contributes to persistent parasitemia and relapsing infection patterns [14,15,16]. Clinical manifestations range from subclinical carriage to severe anemia, immunosuppression, neurological involvement, and reproductive failure, with direct implications for productivity and animal welfare [5,8]. Nevertheless, buffalo-specific immunopathological mechanisms remain incompletely elucidated, and longitudinal studies investigating chronic carriage and relapse dynamics are scarce.

Diagnostic approaches have evolved substantially with the incorporation of molecular tools, including PCR-based assays capable of detecting low parasitemia in chronically infected animals [3,4]. However, field applicability remains limited in many endemic regions due to infrastructural constraints. Therapeutic strategies rely primarily on diminazene aceturate and isometamidium chloride, yet reports of treatment failure and emerging drug resistance particularly in cattle raise concerns about long-term efficacy [8,13,17,18]. Preventive measures are largely dependent on vector control and biosecurity practices, which are challenging to implement sustainably in floodplain and tropical production environments [16]. Furthermore, despite recent advances exploring invariant antigens and novel immunization platforms, no effective vaccine is currently available [19,20].

Several knowledge gaps constrain evidence-based management of *T. vivax* in buffalo herds. First, immunological models specifically designed for water buffaloes are limited, hindering a mechanistic understanding of host tolerance and susceptibility. Second, the epidemiological contribution of buffaloes as reservoirs may be underestimated due to underdiagnosis of low-parasitemia infections and insufficient surveillance in mixed-species systems. Third, buffalo-specific economic impact assessments remain scarce, preventing accurate cost–benefit analyses of integrated control strategies. In light of these limitations, this review provides a comprehensive, species-centered synthesis of *T. vivax* infection in water buffaloes, integrating pathogenesis, epidemiology, diagnosis, and control perspectives. By synthesizing global evidence while utilizing the Amazon biome as a model system for contextualized epidemiological interpretation, this work moves beyond descriptive regional reporting and provides a structured foundation for host-specific research prioritization, translational diagnostics, and sustainable disease management strategies in tropical livestock systems.

## 2. Materials and Methods

This review was conducted as a structured narrative synthesis designed to critically integrate and interpret the available evidence on *Trypanosoma vivax* infection in water buffaloes (*Bubalus bubalis*). Although informed by scoping principles and conceptually aligned with the SANRA (Scale for the Assessment of Narrative Review Articles) framework [21], the primary objective was not to perform a systematic review or meta-analysis, but rather to develop an analytical, host-centered synthesis of heterogeneous experimental, epidemiological, clinical, and molecular data. This work constitutes a structured narrative synthesis guided by transparency principles derived from SANRA [21], but it was not designed as a formal scoping review under PRISMA-ScR methodology [22]. The objective was to construct a host-centered analytical integration of heterogeneous evidence rather than to perform systematic mapping or quantitative aggregation. Therefore, no formal risk-of-bias scoring or critical appraisal instrument was applied. Instead, emphasis was placed on mechanistic interpretation, cross-study comparison, and identification of buffalo-specific knowledge gaps

The literature search was performed between January and December 2025 using PubMed/MEDLINE, Scopus, Web of Science, and SciELO databases. The search strategy combined controlled vocabulary and free-text terms using the Boolean expression (“*Trypanosoma vivax*” OR “*T. vivax*”) AND (“buffalo” OR “water buffalo” OR “*Bubalus bubalis*”). The search window primarily covered publications from 2000 to 2025 in order to capture contemporary advances in molecular epidemiology, diagnostics, immunopathology, and control strategies.

However, seminal experimental studies on infections, published before 2000, have also been included due to their fundamental relevance for understanding buffalo susceptibility, reservoir competence, and transmission dynamics [23,24,25,26]. The foundational studies included provide critical experimental and epidemiological insights into host-specific infection patterns and transmission biology. Eligibility of studies was determined based on direct relevance to buffalo infection, host–parasite interaction, epidemiological dynamics, diagnostic performance, therapeutic implications, or integrated control strategies. Both naturally occurring and experimentally induced infections were considered. Studies exclusively focused on other host species were included only when providing comparative or mechanistic insights applicable to buffalo epidemiology. Conference abstracts without peer review and reports lacking confirmatory diagnostic evidence were not considered.

Data were analyzed qualitatively rather than quantitatively. Emphasis was placed on mechanistic interpretation, cross-regional comparison, identification of conceptual controversies, and delineation of knowledge gaps specific to buffalo production systems. Given the heterogeneity in study design, diagnostic platforms, ecological contexts, and outcome reporting, a quantitative meta-analysis was considered methodologically inappropriate. Instead, evidence was synthesized to construct an integrated analytical framework highlighting host-specific patterns, translational challenges, and research priorities. The main characteristics of the studies included in this structured narrative synthesis are summarized in Table S1, allowing comparative visualization of study design, geographic distribution, diagnostic approaches, and buffalo-specific relevance.

## 3. Etiopathogenesis

*Trypanosoma vivax* is a flagellated protozoan belonging to the class Kinetoplastida, family Trypanosomatidae, genus *Trypanosoma*, and subgenus Duttonella [2,23]. Transmission dynamics differ substantially between cyclical and mechanical systems, with implications for reservoir persistence [3,13]. In the biological cycle, *T. vivax* belongs to the Salivaria section, as the parasite develops in the salivary glands of the tsetse fly (*Glossina* spp.), the main invertebrate host in Africa [3,13,15]. Interestingly, Glossina flies, although central to biological transmission, may also contribute to mechanical spread. In Latin America, however, biting flies such as *Tabanus* spp., *Haematobia irritans* and *Stomoxys calcitrans* are the most important mechanical vectors [3]. Additionally, iatrogenic transmission has been reported, particularly through the reuse of contaminated needles and syringes during medical procedures or vaccination [27,28]. Of special concern in buffalo dairy systems is the frequent use of shared needles for oxytocin administration in lactating buffalo cows prior to or during milking, which represents a critical biosecurity gap.

**Figure 1:**
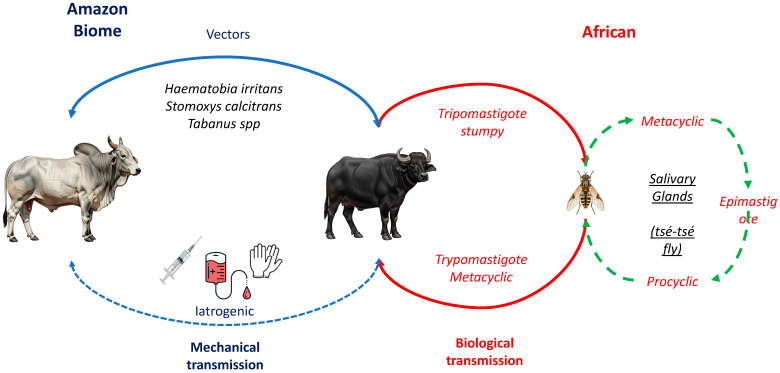
Life cycle of *Trypanosoma vivax* in water buffaloes, highlighting biological transmission by *Glossina* spp. (Africa) and mechanical/iatrogenic transmission by hematophagous flies and contaminated instruments (South America), with emphasis on the Amazon biome. Adapted from Pereira et al. [20].

The life cycle of *T. vivax* (Figure 1) alternates between an invertebrate vector and a vertebrate host [20]. In tsetse flies, the parasite develops exclusively in the proboscis, without undergoing a procyclic stage in the insect midgut and salivary glands [29]. This characteristic distinguishes *T. vivax* from other trypanosomes, such as *T. brucei* and *T. congolense*, which develop as procyclic forms in the tsetse gut [15,26]. Within the proboscis, trypomastigotes differentiate into epimastigotes and subsequently into metacyclic trypomastigotes, the infective stage transmitted to vertebrate hosts via insect bites [13,16]. This restricted developmental cycle may explain why mechanical transmission by other hematophagous flies is feasible in the Americas [30,31]. Once transmitted, metacyclic trypomastigotes enter the host’s bloodstream, where they initially express metacyclic variant surface glycoproteins (mVSGs), providing the first defense against host antibodies [14,15,16]. Inside the mammalian host, the trypomastigotes differentiate into three forms: slender, intermediate, and stumpy [20]; these parasites rapidly transform into bloodstream trypomastigotes, which proliferate by binary fission and disseminate through blood, lymph nodes, and occasionally the cerebrospinal fluid. Their invasion of multiple tissues underlies the diverse clinical signs of animal trypanosomiasis [16,20,32].

In mechanical transmission, vectors remain infective only for a limited period, and thus the frequency of fly bites combined with the level of parasitemia in the reservoir host are decisive factors for successful transmission [13,33]. The prepatent period of *T. vivax* infection varies depending on both the host species and the parasite isolate, and parasitemia often follows irregular daily fluctuations [13]. Another way of mechanical transmission can occur through the use of reused needles or syringes in the herd to collect blood samples or administer medications. This is a common management condition on farms in Brazil, but not recommended. Blood transfusions and the use of tools that scarify the skin of buffaloes also have the potential to transmit *T. vivax*, but this is not a significant epidemiological condition. Reused needles or syringes used across multiple animals can become contaminated, facilitating the spread of *T. vivax*. Research indicates that infection rates can reach 30% via subcutaneous injection, 50% through intramuscular injection, and up to 80% via intravenous injection when contaminated needles are involved. Notably, *T. vivax* can remain viable in certain substances such as foot-and-mouth disease vaccines for up to 20 h, posing a significant risk during veterinary procedures [20].

Despite evidence of susceptibility and chronic carriage, mechanistic immunopathological studies specifically designed for *Bubalus bubalis* remain limited. Most experimental models derive from cattle-based paradigms, potentially overlooking buffalo-specific immune regulation. The molecular basis of tolerance versus susceptibility in buffalo genotypes requires longitudinal investigation integrating transcriptomics and functional immunology. Furthermore, comparative virulence between African and South American strains in buffalo hosts remains insufficiently characterized.

## 4. Epidemiology

### 4.1. Geographic Distribution and Transmission Routes

**Figure 2:**
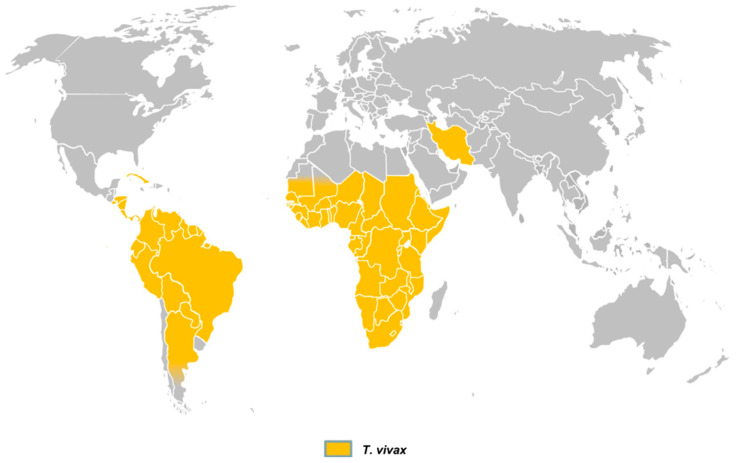
Global distribution of *T. vivax* [2].

*Trypanosoma vivax* is originally from Africa, where its biological cycle involves cyclical transmission by tsetse flies (*Glossina* spp.). However, the parasite has now spread to Latin America and the Caribbean [7,20], where mechanical transmission represents the critical factor for its dissemination and persistence in regions without tsetse flies [13,34]. In a comprehensive review on the diagnosis of animal trypanosomoses, Desquesnes et al. [2] presented the global distribution of *T. vivax* (Figure 2), which currently includes most countries in South and Central America, large areas of sub-Saharan Africa, and, more recently, reports from Iran. In Central and South America, mechanical transmission is mainly associated with biting flies such as *Tabanus* spp., *Stomoxys calcitrans* and *Haematobia irritans* [3,35]. Although *T. vivax* DNA has also been detected in ticks (*Amblyomma cajennense*, *Rhipicephalus microplus*) and in the buffalo louse *Haematopinus tuberculatus*, there is no scientific evidence to date that these ectoparasites play an epidemiological role in transmission and in the state of Pará, Brazil, especially on Marajó Island, high infestation on buffaloes by this louse is common [4].

### 4.2. Reservoir Role and Endemic Stability

This parasite infects both wild and domestic ungulates, including buffaloes, cattle, sheep, and goats [13,36], as well as camels, donkeys and even suids [2,35]. In Africa, several wild ruminants, especially the African buffalo (*Syncerus caffer*), are considered important reservoirs. In contrast, no wild reservoirs have been reported in South America [8,35]. Among domestic species, buffaloes often act as healthy carriers, similar to cattle and goats, particularly when chronically infected [8,37]. These changes in parasitemia are associated with the host’s immune reaction, the antigenic shifts in the surface variant glycoproteins of trypanosomes, seasonal variations and parasite traits [38,39]. These asymptomatic animals are epidemiologically relevant because they can silently disseminate *T. vivax* into herds and regions where no clinical cases are apparent [3,35], principally from endemic regions without prior quarantine or performing diagnostic tests. Pérez et al. [5] further demonstrated that buffaloes and cattle may present high infection rates, acting as subclinical carriers, and that co-infections with other trypanosomes, such as *T. theileri*, can occur.

### 4.3. Seasonality, Risk Factors, and Prevalence Heterogeneity

Several South American countries, including Venezuela, Brazil, Colombia, and Bolivia, are now regarded as endemic areas for *T. vivax* [5,8]. Amazonian lowlands, Venezuelan Llanos and the Brazilian Pantanal are considered regions of enzootic stability of *T. vivax* infections [40]. In the Brazilian Amazon, particularly in the Lower Amazon region and Marajó Island, the combination of buffalo and cattle rearing under floodplain (*várzea*) and upland pasture systems at high stocking densities creates favorable conditions for intense exposure to hematophagous flies, maintaining trypanosomiasis endemicity [5]. Flooded environments in *várzea* pastures further increase vector density, thereby reinforcing transmission [16]. Under such endemic conditions, buffaloes often harbor chronic, low to moderate parasitemia, controlled by continuous immune exposure, characterizing a state of enzootic stability [5,8,27,41,42,43]. Conversely, the climate variations seen in recent years may disturb this enzootic balance and trigger outbreaks in endemic areas [43]. A persistent controversy concerns whether buffaloes act primarily as maintenance reservoirs or incidental spillover hosts. Evidence from African experimental transmission studies suggest sustained infectivity under cyclical transmission (24–26), whereas South American systems dominated by mechanical transmission may alter reservoir efficiency. Direct comparative transmission efficiency studies in mechanical vector systems remain scarce. (Table 1) [3,4,5,8,36,43]. The highest prevalence values are in the rainy season (*p* < 0.05), when biting flies are highly abundant, but there are reports of outbreaks in the dry season, in areas with severe drought (stress) [8].

Interestingly, Serra et al. [3] observed no statistical differences in antibody detection rates against *T. vivax* across buffalo age groups, which contrasts with cattle, where older animals are generally more susceptible [44,45,46]. Similarly, no significant age and sex-related differences were described in buffaloes [47], but only one report indicates that younger buffaloes infected by *Trypanosoma* sp., under 12 months of age, are more severely impacted [48]. No differences between age and sex are common in other species [40,49,50]. *T. vivax* prevalence in buffaloes varies considerably but tends to increase during rainy seasons, correlating with the greater transmission of *T. vivax* due to the increase in the population of hematophagous flies during the rainy season and higher animal density [8,31,41]. Although many infections remain asymptomatic, clinical disease may be triggered or reactivated by stressors such as thermal stress, poor nutrition, pregnancy, and lactation, or by co-infections with pathogens such as *Babesia* spp. and *Anaplasma* spp. [5,8]. These stressors compromise immune control, facilitating parasitemia escalation and leading to severe or even fatal outcomes of trypanosomiasis in buffalo herds [8].

On the other hand, certain cattle breeds have been reported to possess natural resistance to trypanosomiasis referred to as trypanotolerant including *Bos taurus* N’Dama, Muturu, and Dahomey, predominantly found in West Africa. In regions with a high risk of infection, these trypanotolerant cattle are able to maintain growth and reproductive performance [13]. In African buffaloes, studies have shown that resistance to trypanosomiasis developed through evolutionary adaptation, involving the production of specific antibodies against variant surface glycoproteins (VSG) and the generation of trypanocidal H_2_O_2_ molecules that lead to the elimination of the parasite from the bloodstream [51,52]. Additionally, a study examining the expression patterns of IFN-γ and miRNA-125b in dairy buffaloes (*B. bubalis*) with and without *T. vivax* infection revealed distinct gene expression profiles between infected and uninfected genotypes. Buffaloes positive for *T. vivax* carrying the AA and GA genotypes appeared more susceptible to infection, exhibiting elevated IFN-γ levels and reduced miRNA-125b expression. In contrast, uninfected buffaloes with the GG genotype showed increased resistance to *T. vivax*, characterized by higher expression of both IFN-γ and miRNA-125b, likely due to the G allele enhancing their regulatory interaction [52].

Reported prevalence variability across regions (1.9% to >80%) must be interpreted cautiously. Differences in diagnostic platforms (serology vs. PCR), sampling strategies (herd-level vs. outbreak investigations), seasonal timing, and ecological context (mechanical vs. cyclical transmission systems) likely account for much of this heterogeneity. Cross-sectional designs predominate, limiting inference regarding temporal dynamics and chronic carriage stability in buffalo populations. A local example from the municipality of Soure on Marajó Island is provided in the Supplementary Materials. The methodology and results are detailed and illustrated with figures in the Supplementary Materials (Figure S1 and Table S1), where they serve as illustrative examples rather than central evidence and do not form part of the evidence base for this review, thereby preserving consistency in the review structure.

### 4.4. Economic Impact and Evidence Gaps

Beyond the detrimental effects on animal health, trypanosomiasis generates substantial economic losses through reduced body condition, decreased milk yield, reproductive impairment, and increased mortality [53]. In cattle, annual losses attributed to trypanosomiasis in Africa are estimated at approximately USD 5 billion, with an additional USD 30 million spent annually on treatments [7,54,55]. In Brazil, particularly in the Pantanal wetland an ecosystem with seasonal flooding comparable to Marajó Island losses exceeding USD 160 million have been estimated in bovine production systems [7,56].

However, robust buffalo-specific economic impact data remain scarce, and this absence represents a significant research gap. Most available estimates derive from cattle-based production models and are frequently extrapolated to buffalo systems without incorporating species-specific productivity parameters, management structures, or pharmacological considerations. Although outbreaks of *T. vivax* in buffalo herds have been associated with marked clinical deterioration and productivity losses [5,8], structured economic assessments quantifying direct and indirect costs in buffalo-dominated systems are lacking. Consequently, while analogies with bovine systems raise legitimate concern particularly for small- to medium-scale producers who predominate in Amazonian buffalo husbandry they must be interpreted cautiously to avoid overestimation or unsupported generalization.

This knowledge gap highlights the need for targeted cost–benefit analyses integrating buffalo-specific milk yield reduction, reproductive losses, treatment expenditures, and mortality rates under endemic mechanical transmission systems. Furthermore, buffalo-specific transmission coefficients under mechanical vector pressure have not been experimentally quantified [5,8], limiting the precision of epidemiological–economic modeling. Longitudinal cohort studies evaluating parasitemia dynamics, vector density, and seasonal hydrological patterns are required to refine outbreak prediction and estimate the true economic burden of *T. vivax* in buffalo-dominated tropical production systems.

## 5. Clinical Signs and Necropsy Findings

In endemic areas of *Trypanosoma vivax*, water buffaloes may remain asymptomatic or develop a chronic course of infection with insidious progression of clinical signs. The most common manifestations include intermittent fever, progressive weight loss, anemia, and reproductive disorders such as abortion [5,8,13]. The anemia, according to Guegan et al. [57], implying an ex vivo assay to measure erythrophagocytosis throughout infection, demonstrated that trans-sialidase enzymes, released in the early stages, induce desalination of erythrocytes, leading to their phagocytosis and contributing to anemia. Although anemia is consistently reported, the relative contribution of immune-mediated erythrophagocytosis versus direct parasitic effects may differ between cattle and buffaloes. Controlled comparative hematological kinetics studies are lacking [5,8,13]. Furthermore, the same authors showed that erythrophagocytosis is responsible for the initial significant decline in hematocrit in the acute phase of infection. Concomitant stress factors, including nutritional deficits or co-infections, have been reported to exacerbate the clinical course and increase the likelihood of symptomatic disease [8]. Importantly, the clinical presentation in buffaloes is generally similar to that observed in cattle and other ruminants, as seen in sheep, indicating comparable patterns of disease expression across species [13,58,59].

A well-documented outbreak described by Garcia et al. [8] in buffaloes from the Venezuelan Llanos highlighted the occurrence of neurological involvement, which is less frequently reported in bovines. The most severely affected animals exhibited depression, muscle tremors, and severe ataxia, often characterized by dragging of the forelimbs. In this outbreak, the mortality rate reached 7% of the herd, considered high for trypanosomiasis, and cases persisted for nine months after the onset of clinical disease. The prolonged impact was likely aggravated by nutritional stress, stable fly infestations, and lack of preventive health measures, such as strategic deworming and ectoparasite control, which may have favored the severity of clinical signs and contributed to the observed mortality.

Regarding necropsy findings, carcasses of buffaloes clinically affected by *T. vivax* trypanosomiasis are frequently described as edematous and markedly anemic [13,60]. In cattle, postmortem lesions are generally nonspecific and may include petechiae on serous membranes, lymphadenomegaly, splenomegaly, serous atrophy of fat, and evidence of systemic anemia [16]. These observations highlight the need for confirmatory laboratory testing, as necropsy findings alone are not pathognomonic for the disease but may support clinical suspicion when correlated with epidemiological context and diagnostic results. Standardized clinical scoring systems for buffalo trypanosomiasis have not been developed. Prospective studies distinguishing acute, chronic, and subclinical trajectories in buffaloes are required to refine case definitions and improve early detection.

## 6. Diagnosis

### 6.1. Parasitological and Concentration Techniques

**Figure 3:**
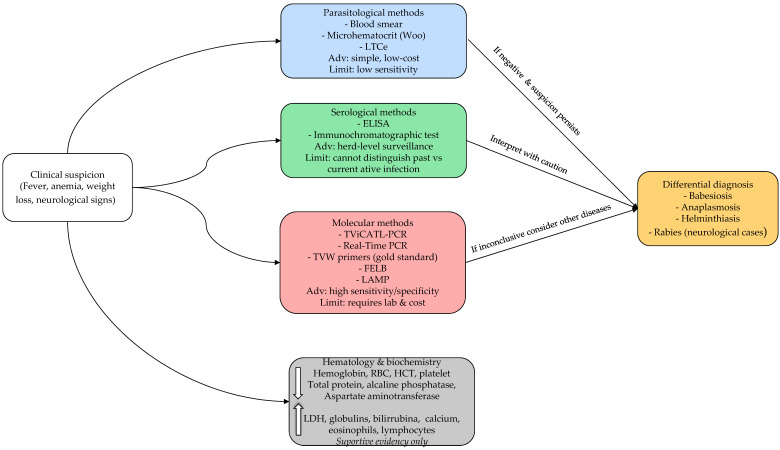
Practical diagnostic workflow for the diagnosis of *Trypanosoma vivax* in water buffaloes, integrating clinical suspicion with parasitological, serological, molecular, and hematological approaches and highlighting differential diagnoses.

The diagnosis of trypanosomiasis caused by *Trypanosoma vivax* in water buffaloes can be established through a combination of clinical examination and parasitological, serological, and molecular methods (Table 2 and Figure 3) [2,5]. Among parasitological tests, the blood smear remains the most widely used due to its simplicity, especially by the Woo technique [60,61], field applicability, and low cost. The first record of *T. vivax* in the Brazilian Amazon was reported through blood smears from buffaloes on Marajó Island in 1972 by Shaw and Lainson [12]. Peripheral blood samples collected from the tail base or ear tip are recommended for buffaloes [3]. However, this method has significant limitations in terms of sensitivity and specificity, particularly in animals with low parasitemia or in asymptomatic carriers [3,4]. While PCR-based assays demonstrate high sensitivity, field deployment remains limited. The critical question is not analytical sensitivity alone but diagnostic accessibility within buffalo production systems characterized by limited infrastructure. Validation of portable platforms such as LAMP under true field conditions remains a priority. As an additional option with the use of peripheral blood samples, the “Lysis and Concentration Technique” (LCTe) enhances the chances of visualization in low parasitemia, because enhances the concentration of hemoprotozoa by eliminating red blood cells. Moreover, LCTe is highly cost-effective and simple to put both in laboratories and in field settings [62].

### 6.2. Serological Surveillance

Serological techniques provide higher sensitivity in herd-level surveillance. Serra et al. [3] reported that 79.31% (92/116) of buffaloes were seropositive by indirect enzyme-linked immunosorbent assay (iELISA), while 76.72% (89/116) tested positive with an immunochromatographic assay (Imunotest^®^). These findings demonstrate that buffaloes are frequently exposed to *T. vivax*, even at parasitemia levels undetectable by blood smears. Furthermore, antibody responses may persist for extended periods after infection, allowing detection even in animals with transient or subclinical infections [48,49]. The iELISA has been extensively applied in cattle and is considered highly sensitive, particularly in naturally infected animals [64].

### 6.3. Molecular Diagnostics and Field-Adapted Platforms

Molecular methods have advanced the diagnosis of *T. vivax* in buffaloes and their vectors [3,4] and are effective in identifying active infections during the chronic stage, when low parasitemia levels limit the effectiveness of traditional parasitological methods [43]. Currently, three buffalo-derived sequences are deposited in GenBank (accessions OR339796, MK801872, MK801874), showing close phylogenetic relationships [3]. Polymerase chain reaction (PCR) is regarded as one of the most sensitive and specific diagnostic tools, requiring only small volumes of blood and being applicable to samples preserved at room temperature [5,8,65]. Furthermore, real-time PCR can be applied to hosts and vectors with high sensitivity and specificity [1]. Despite its advantages, false-negative outcomes may still arise when parasitemia is extremely low, as seen in chronic infections, when excessive DNA is added to the PCR, or in the presence of inhibitory substances. Conversely, false-positive results can occur due to contamination from other positive samples [1,43]. As disadvantages, PCR requires well-equipped laboratories, trained personnel, and high-quality DNA preparations with appropriate primers. Although more costly than parasitological methods, PCR offers substantial improvements in diagnostic accuracy [2]. Specific assays include the TviCATL-PCR, described by Cortez et al. [66], and the Fluorescent Fragment Length Barcoding (FFLB) test, both of which can successfully detect *T. vivax* in buffaloes, even in ethanol-preserved samples without refrigeration [5]. In addition, Desquesnes et al. [2] highlighted the TVW primers as the gold standard for *T. vivax* molecular detection.

Additionally, the Loop-Mediated Isothermal Amplification (LAMP) is notable as methodology using nucleic acid isothermal amplification assays in the detection of *T. vivax* in blood samples of buffaloes naturally infected in the Brazilian Amazon, indicating that it represents a practical alternative to conventional molecular methods, with promising applications in epidemiological surveillance and effective clinical diagnosis [43]. In LAMP, the isothermal amplification process permits diagnosis using basic equipment, such as a dry block heater or water bath, making it suitable for use in field diagnostics [63]. Nevertheless, additional research is necessary to optimize LAMP protocols, simplifying result interpretation and supporting their integration into routine laboratory practice [43]. Comparative diagnostic accuracy studies specifically in buffaloes are limited. The performance of molecular assays validated in cattle cannot be assumed equivalent. Cost-effectiveness analyses of herd-level screening strategies in buffalo-dominated systems are urgently needed.

Beyond conventional PCR platforms, additional high-sensitivity approaches have been explored to improve detection of *T. vivax* in low-parasitemia infections. The mini–Anion Exchange Centrifugation Technique (mAECT), although parasitological rather than molecular, increases diagnostic sensitivity by concentrating viable trypanosomes through anion-exchange separation, outperforming microhematocrit-based methods in subpatent infections while maintaining high specificity due to direct parasite visualization [2,61]. However, mAECT requires centrifugation equipment, specific columns, and trained personnel, which limits its deployment in remote Amazonian production systems. At the molecular level, Spliced-Leader qPCR (SL-qPCR) provides very high sensitivity and specificity by targeting parasite RNA transcripts, thereby offering stronger evidence of active infection compared to DNA-based PCR assays [2]. This feature may be particularly advantageous for detecting chronic carriers and monitoring post-treatment parasitemia. In contrast, LAMP offers lower infrastructural requirements and greater field adaptability but may present variability in interpretation and standardization, particularly under true field conditions [43]. Thus, SL-qPCR maximizes analytical sensitivity in laboratory settings, whereas LAMP optimizes operational feasibility in resource-limited environments. These complementary characteristics support the implementation of a tiered diagnostic approach in buffalo-dominated systems, in which field-adapted screening methods are combined with high-sensitivity confirmatory assays according to epidemiological context and available infrastructure.

### 6.4. Hematological and Biochemical Supportive Findings

Hematological and biochemical alterations further support the diagnosis of *T. vivax* trypanosomiasis in buffaloes. Common findings include severe reductions in hemoglobin concentration, hematocrit, red blood cell counts, creatinine, urea, and alkaline phosphatase, along with increased leukocyte counts, lactate dehydrogenase activity, globulin levels, and both total and indirect bilirubin [67,68,69,70]. In another way, natural *Trypanosoma* spp. in water buffaloes’ infection resulted in statistically significant differences (*p* < 0.05), decreasing red blood cells, hemoglobin, pack cell volume, platelets and reduced concentrations of total protein, alkaline phosphatase, and aspartate aminotransferase. On the other hand, increases in eosinophils, lymphocites and serum calcium [71].

Despite these advances, several diagnostic challenges persist, such as: the need for adequately trained technical teams and fully equipped laboratories; limited availability of standardized commercial reagents; restricted access to advanced laboratory tests in endemic regions; and high costs of molecular assays [2]. Therefore, progress in the diagnosis of buffalo trypanosomiasis relies on the development of more affordable, rapid, and field-adapted methods, particularly in resource-limited endemic countries.

### 6.5. Differential Diagnosis

Differential diagnosis is also essential, as *T. vivax* infections share clinical similarities with other diseases. During acute febrile phases, trypanosomiasis should be distinguished from babesiosis and anaplasmosis, since *T. vivax* infections rarely produce jaundice or hemoglobinuria, and parasitological examination allows differentiation [13]. In chronic forms characterized by emaciation, lymphadenopathy, and absence of fever, the main differentials are helminthiases, which can be confirmed through parasitological testing. For neurological cases, rabies must be carefully excluded, given its public health importance. Unlike trypanosomiasis, rabies is not associated with anemia or weight loss, and its confirmation requires specific laboratory techniques such as direct immunofluorescence, PCR, mouse inoculation, or histopathology.

## 7. Control and Prophylaxis

### 7.1. Principles of Integrated Control in Buffalo Herds

Global control programs for trypanosomiasis generally integrate three main pillars: vector control, diagnosis, and treatment. In the context of *T. vivax*, disease management in buffaloes requires restricting the movement of clinically affected animals, systematic herd monitoring, and timely therapeutic intervention. Supportive measures are also critical to improve the efficacy of trypanocidal drugs, such as avoiding animal transportation and pasture changes during the acute phase, while ensuring adequate nutrition and balanced supplementation [5,8,13].

### 7.2. Chemotherapy, Treatment Failures, and Resistance

The principal trypanocidal drugs used in buffaloes, cattle, sheep, and goats are diminazene aceturate and isometamidium chloride [8,13,64]. In Brazil, these two compounds are the only licensed trypanocides authorized by the Ministry of Agriculture, Livestock and Supply (MAPA) [60]. Nevertheless, intensive use has already led to reports of drug resistance [17,18]. Another option, although less frequently employed, is homidium bromide or chloride [16]. In an outbreak of *T. vivax* in Venezuela [8], affected buffalo herds were successfully treated with isometamidium chloride (1.0 mg/kg body weight, intramuscularly), in combination with supportive therapy consisting of multivitamin supplementation and hematinic drugs. Treatment reduced mortality and prevented new symptomatic cases, confirming both the etiological role of *T. vivax* and the effectiveness of the drug in that context. In addition, the latest trypanocide to be developed is melarsomine dihydrochloride. Nonetheless, this trypanocide causes nervous signs in buffaloes (0.75 mg/kg body weight, intramusculary), in a transient side effect [67]. The sustainability of chemotherapeutic reliance is increasingly questioned. Although resistance is well documented in cattle, systematic resistance surveillance in buffalo populations remains largely unexplored. Extrapolation from bovine data may underestimate emerging risk. Buffalo-specific pharmacokinetic data for trypanocides remain limited. Drug metabolism differences between cattle and buffaloes may influence therapeutic efficacy. Integrated vector–host modeling approaches adapted to floodplain ecosystems are required to develop sustainable control strategies. A synthesis of these treatments is described in Table 3.

Although trypanocide resistance has been extensively documented in African bovine systems, particularly for diminazene aceturate and isometamidium chloride [17,64], the strength of direct evidence supporting resistance in Trypanosoma vivax infecting water buffaloes remains comparatively limited. Most reports of reduced therapeutic efficacy derive from cattle populations, where repeated drug exposure, underdosing, and absence of structured surveillance have contributed to resistance selection. In buffaloes, published data are largely confined to outbreak response scenarios demonstrating clinical improvement after treatment [8], without systematic post-treatment parasitological monitoring or pharmacokinetic validation. Consequently, extrapolation from bovine systems to buffalo herds while biologically plausible must be interpreted cautiously. In endemic Amazonian systems characterized by mechanical transmission, high vector density, and frequent animal movement between floodplain pastures, subclinical chronic carriers may persist after treatment, potentially sustaining parasite circulation even in the absence of overt therapeutic failure. The absence of structured resistance surveillance and buffalo-specific pharmacokinetic data therefore represents a critical vulnerability for control programs. Without species-specific evidence, reliance on bovine-derived treatment paradigms may underestimate emerging resistance risks and compromise long-term sustainability of trypanosomiasis control in buffalo-dominated tropical production systems [2].

### 7.3. Biosecurity and Iatrogenic Transmission Prevention

From a preventive perspective, quarantine of newly introduced animals, although not a widespread practice, is an essential biosecurity measure to avoid introduction of *T. vivax* into non-endemic regions. Likewise, proper handling of needles and syringes during vaccination or drug administration is fundamental to prevent iatrogenic transmission [3]. In West Africa, the presence of so-called trypanotolerant breeds of cattle, particularly the N’Dama, has been reported as an important strategy to reduce trypanocide usage [16]. However, to date, no trypanotolerant breeds have been described among water buffaloes.

### 7.4. Vector Control in Floodplain and Tropical Systems

Vector control requires consideration of the target species, its ecological niche, climatic conditions, and agroecological context [16]. Holmes [16] describes several environmentally acceptable tactics for tsetse fly control and eradication, including: the Sequential Aerosol Technique (SAT), involving four to five applications of ultra-low volume, non-persistent insecticides by GPS-guided aircraft, with excellent results in savanna ecosystems; the live bait technique, consisting of insecticide application to livestock via spraying, which also protects against other ectoparasites; the artificial bait system, involving insecticide-impregnated traps or target fabrics in blue and black, capable of suppressing up to 95% of fly populations; and the sterile insect technique, in which sterilized males are released to interrupt reproduction, used when other strategies fail to achieve sufficient suppression.

### 7.5. Vaccine Prospects and Translational Challenges

In parallel, research efforts have advanced toward the development of vaccines. A recent study reported a candidate vaccine targeting the invariant surface glycoprotein IFX of *T. vivax*, which induced protective immunity in a murine model [19]. Nonetheless, vaccine development against trypanosomes faces significant obstacles, including parasite antigenic variation, host immunosuppression, and the lack of robust experimental models. Even so, current research highlights promising avenues, such as the identification of invariant proteins and the use of innovative technologies including mRNA vaccines and gene editing platforms [3].

## 8. Methodological Constraints and Evidence Gaps

The current body of literature is characterized by cross-sectional predominance, heterogeneous diagnostic methodologies, limited buffalo-specific longitudinal cohorts, and absence of standardized prevalence reporting criteria. These limitations restrict robust estimation of reservoir competence and economic impact in buffalo-dominated systems. Future studies should prioritize longitudinal parasitemia monitoring, comparative diagnostic accuracy validation, and pharmacokinetic assessment of trypanocides in buffaloes.

## 9. Conclusions

This structured narrative review repositions *Trypanosoma vivax* infection in water buffaloes (*Bubalus bubalis*) within a host-centered analytical framework, moving beyond the traditional cattle-dominant paradigm that has historically shaped the interpretation of animal trypanosomiasis. By integrating evidence from cyclical and mechanical transmission systems across Africa and the Americas, the present synthesis demonstrates that buffaloes are not incidental hosts but epidemiologically competent and clinically susceptible animals whose role in parasite maintenance and transmission requires explicit consideration. The comparative interpretation of global data highlights that buffalo infection dynamics are shaped by transmission ecology, herd structure, and vector pressure, rather than by geography alone. Case-based contextualization using high-density buffalo production systems under mechanical transmission illustrates how ecological and management factors interact to sustain chronic carriage and sporadic outbreaks. Importantly, low-parasitemia persistence, diagnostic limitations in subclinical animals, and the emerging concern of trypanocide resistance represent converging challenges for sustainable control.

Despite progress in molecular diagnostics and immunological research, buffalo-specific mechanistic studies remain limited. Key priorities include longitudinal investigations of chronic infection kinetics, pharmacokinetic evaluation of trypanocides in buffaloes, transmission modeling under mechanical vector systems, and development of field-adapted diagnostic platforms suitable for tropical production environments. By consolidating heterogeneous evidence into a structured, buffalo-centered perspective, this review provides a conceptual foundation for targeted surveillance, translational research, and integrated control strategies adapted to tropical livestock systems.

## Figures and Tables

**Table 1:** Prevalence of *T. vivax* infection in buffaloes by region, farm, diagnostic method and main risk factors.

Country/Region	Prevalence	Diagnosis Method	Risk Factors	Observations	Reference
Brazil, Amazon	79.31% (92/116)/76.72% (89/116)	iELISA/Imunotest^®^	Absence of quarantine for newly introduced buffaloes	No differences: male x female, age and sex (*p* < 0.05)	[3]
Brazil, Amazon	1.93% (12/621)	PCR	High animal concentration in some cases	60 farms collected	[4]
Brazil, Amazon	28.08% (25/89)/59.60% (53/89)	TviCATL-PCR/FFLB	Overstocked dry land farms with buffalo and cattle exposed to many biting flies in one year	No differences in the *T. vivax* infection rates between cattle and buffaloes	[5]
Brazil, Amazon	4% (6/150)/38% (57/150)/83.33% (125/150)	PCR/Real Time PCR/LAMP	History of ectoparasites infestation	Samples: buffaloes and cattle	[43]
Brazil and Paraguay, Pantanal	34.88% (15/43)	PCR	Rainy season (high biting flies density)	Mixed infections (*Trypanozoon + T. vivax*) detected by PCR were: 6.9% (3/43)	[36]
Venezuela, Llanos	32.11% (67/293)/30.4% (35/115-outbreak)	TviCATL-PCR	Higher prevalence values in the rainy season (*p* < 0.05); outbreak in an area with a severe drought (stress)	5.3–47.8% of infected buffalo in different farms	[8]

**Table 2:** Comparison of the applicability of the main diagnostic tests used in buffaloes.

Type	Sensitivity/ Specificity	Laboratory Requirements	Costs	Field Applicability	Applications	References
Blood smear	Medium Specificity Low sensitivity	Simple	Low	Yes	Symptomatic cases/acute phase/individual cases	[3,43]
Microhematocrit (Woo)	Low	Simple	Low	Yes	Symptomatic cases/acute phase/individual cases	[43,61]
LCTe	Low	Simple	Low	Yes	Symptomatic or asymptomatic cases/acute phase/individual cases	[62]
iELISA	High	Advanced	High	No	Symptomatic or asymptomatic cases/acute or chronic phase/herd cases	[3]
PCR	High	Advanced	High	No	Symptomatic or asymptomatic cases/acute or chronic phase/individual or herd cases	[1,43]
TviCATL/TVW	High	Advanced	High	No	Symptomatic or asymptomatic cases/acute or chronic phase/individual or herd cases	[5]
LAMP	High	Advanced	Medium	Yes	Symptomatic or asymptomatic cases/acute or chronic phase/individual or herd cases	[43,63]
mAECT (mini-Anion Exchange Centrifugation Technique)	High	Advanced	High	Yes	Symptomatic or asymptomatic cases/chronic phase/individual or herd cases	[61]
Spliced-Leader qPCR (SL-qPCR)	Very High	Advanced	High	No	Symptomatic or asymptomatic cases/acute or chronic phase/individual or herd cases	[43]

**Table 3:** Summary of chemotherapeutic options reported for *Trypanosoma vivax*, with emphasis on water buffaloes.

Drug	Dosage ^a^	Route	Indication	Species	Observation	References
Isometamidium chloride	1.0	IM ^b^	Curative Prophylactic	Buffaloes	Reduction in mortality and new cases	[8]
Isometamidium chloride	1.0	IM	Curative Prophylactic	Cattle	Suggestion of resistance (continuous detection of anti-*T. vivax* IgG antibodies)	[64]
Diminazene aceturate	7.0	IM	Curative	Ruminants	Mostly used. Worldwide resistance	[67]
Melarsomine dihydrochloride	0.75	IM	Not informed	Buffaloes	Causes nervous signs in a transient side effect	[67]

^a^ mg/kg body weight, ^b^ Intramusculary.

## Data Availability

The original contributions presented in the study are included in the article/Supplementary Materials; further inquiries can be directed to the corresponding author.

## Supplementary Materials

The following supporting information can be downloaded at: https://www.mdpi.com/article/10.3390/pathogens15030273/s1, Figure S1: A 1.5% agarose gel demonstrating the presence of *T. vivax*. Lane L corresponds to the 100 base pair molecular weight marker (100 bp DNA Ladder). Lanes 1 to 72 are PCR products (amplicons) derived by DNA from blood samples corresponding to 72 different buffaloes. Lane C+ corresponds to the positive control, which represents the amplicon formed by DNA from a buffalo blood sample known to be positive for *T. vivax*. Lane C- corresponds to the negative control, which represents the amplicon formed by ultrapure sterilized water instead of a sample DNA. Lane 46 represents the buffalo sample positive for *T. vivax*, amplifying at the 177 bp position on the agarose gel, similar to C+; Table S1. Main characteristics of studies included in the structured narrative synthesis of *Trypanosoma vivax* infection in water buffaloes (*Bubalus bubalis*).
