# Supplementary material for: Trypanosoma vivax in Water Buffaloes (Bubalus bubalis): A Host-Centered Synthesis of Pathogenesis, Epidemiology, Diagnosis, and Integrated Control with Implications for Tropical Production Systems
Source: Pathogens. 2026 Mar 3;15(3):273. doi: 10.3390/pathogens15030273 (PMC13029234; doi:10.3390/pathogens15030273)
Supplement: Supplementary file 1 [file pathogens-15-00273-s001.zip › pathogens-4123665-supplementary.pdf]

Table S1. Main characteristics of studies included in the structured narrative synthesis of *Trypanosoma vivax* infection in water buffaloes (*Bubalus bubalis*).

Author (Year)/Reference number	Country/Region	Study Type	Host Focus	Diagnostic / Methodological Approach	Main Contribution	Buffalo-Specific Relevance
**Shaw & Lainson (1972)/[12]**	Brazil	Epidemiological report	Livestock	Parasitological	Early report of *T. vivax* in Brazil	Historical introduction context
**Woo & Rogers (1974)/[61]**	–	Diagnostic evaluation	Livestock	Haematocrit centrifuge technique	Diagnostic sensitivity limitations	Underestimation of low parasitemia
**Lanham et al. (1981)/[23]**	Brazil (Amazon)	Experimental/diagnostic	Buffalo	Anion exchange separation	Detection of subpatent infection	Demonstrated chronic low parasitemia
**Dwinger et al. (1986)/[26]**	Africa	Experimental infection	Buffalo	Cyclical transmission	Host susceptibility variation	Buffalo competence under tsetse system
**Moloo et al. (1993)/[25]**	Africa	Reservoir comparison	Buffalo vs cattle	Transmission studies	Reservoir competence	Buffalo as reservoir
**Moloo et al. (1999)[24]**	Africa	Sequential infection	Buffalo	Experimental	Multi-trypanosome dynamics	Ecological co-infection insight
**Dávila et al. (2003)/[36]**	Brazil	Molecular epidemiology	Livestock	PCR	Cryptic epidemiology	Diagnostic advancement
**Garcia et al. (2006)/[41]**	Venezuela	Molecular detection	Buffalo	PCR	Natural infections	Buffalo confirmation
**Desquesnes & Dia (2004)/[31]**	Africa	Mechanical transmission	Cattle	Tabanid transmission	Vector competence	Mechanistic model applicable to buffalo
**Garcia et al. (2005)/[65]**	Venezuela	Molecular characterization	Buffalo	PCR	Parasite detection	Buffalo-specific molecular data
**Tamasaukas et al. (2006)/[47]**	Venezuela	Field report	Buffalo	Parasitological	Farm-level infection	Clinical evidence

**Hilali et al. (2006)/[68]**	–	Clinical pathology	Buffalo	Hematology	Hematological impact	Comparative pathology
**Garcia et al. (2016)/[8]**	Venezuela	Cross-sectional	Buffalo	Serology + PCV	Clinical association	Anaemia link
**Osório et al. (2008)/[13]**	Review	General	Multiple	Review	Biology & epidemiology	Contextual foundation
**Cortez et al. (2009)/[66]**	Africa/S.A.	Molecular	Livestock	Cathepsin gene	Diagnostic implications	Marker development
**Desquesnes et al. (2009)/[33]**	–	Mathematical model	Cattle	Transmission modeling	Mechanical transmission model	Applicable to buffalo systems
**Dayo et al. (2010)/[45]**	Burkina Faso	Cross-sectional	Cattle	Parasitological	Prevalence	Comparative context
**Monzón et al. (2010)/[60]**	Argentina	Field report	Buffalo	Parasitological	Buffalo outbreak	Direct evidence
**Galiza et al. (2011)/[58]**	Brazil	Clinical outbreak	Sheep	Pathology	CNS lesions	Pathogenic potential
**Adam et al. (2012)/[46]**	Ghana	Cross-sectional	Cattle	Multi-platform	Prevalence heterogeneity	Comparative insight
**Fikru et al. (2012)/[49]**	Ethiopia	Cross-sectional	Cattle	Molecular	Tsetse/non-tsetse areas	Transmission ecology
**Angara et al. (2012)/[55]**	–	Economic review	Livestock	Review	Economic impact	Gap in buffalo economics
**Baker et al. (2013)/[17]**	–	Drug resistance review	*T. brucei*	Molecular	Resistance mechanisms	Mechanistic inference
**Guegan et al. (2013)/[57]**	–	Pathogenesis	Livestock	Erythrophagocytosis	Anaemia mechanism	Pathogenic insight
**Garcia et al. (2014)/[41]**	Africa/S.A.	Microsatellite	Livestock	Population genetics	Clonal propagation	Epidemiological inference

**Rodrigues et al. (2015)/[35]**	Brazil	Carrier study	Donkeys	PCR	Healthy carriers	Reservoir ecology
**Garcia et al. (2003)/[70]**	Venezuela	Outbreak	Buffalo	Clinical + PCR	Wasting disease	Direct buffalo outbreak
**Berthier et al. (2016)/[37]**	–	Immunological review	Multiple	Review	Tolerance mechanisms	Chronic carriage relevance
**Ooi et al. (2016)/[29]**	–	Vector biology	Tsetse	Developmental biology	Life cycle	Transmission dynamics
**Morrison et al. (2016)/[6]**	Review	Multiple	Review	Clinical relevance	Understudied hosts	Buffalo neglected host
**Pereira et al. (2020)/[38]**	Molecular	T. vivax	Genomics	Antigen variation	Vaccine relevance	
**Pérez et al. (2020)/[5]**	Brazil	Cross-sectional	Buffalo	PCR	High infection rate	Amazon evidence
**Dyonisio et al. (2021)/[4]**	Amazon	Molecular	Buffalo	PCR	Prevalence in ectoparasites	Vector involvement
**Castilho Neto et al. (2021)/[64]**	Brazil	Treatment follow-up	Cattle	Drug response	Post-treatment dynamics	Pharmacological inference
**Autheman et al. (2021)/[19]**	–	Vaccine study	Experimental	Immunization	Protective antigen	Future control
**Serra et al. (2024)/[3]**	Maranhão	Molecular/serology	Buffalo	PCR + serology	Buffalo infection confirmation	Direct evidence
**Barros Moura et al. (2024)/[43]**	Amazon	Diagnostic comparison	Buffalo	PCR vs qPCR vs LAMP	Diagnostic sensitivity	Low parasitemia detection
**Garcia et al. (2024)/[34]**	South America	Mitochondrial genomics	*T. vivax*	Genomics	Mechanical adaptation	Evolutionary adaptation
