# Supplementary figures and images for: Trypanosoma vivax in Water Buffaloes (Bubalus bubalis): A Host-Centered Synthesis of Pathogenesis, Epidemiology, Diagnosis, and Integrated Control with Implications for Tropical Production Systems
Source: Pathogens. 2026 Mar 3;15(3):273. doi: 10.3390/pathogens15030273 (PMC13029234; doi:10.3390/pathogens15030273)

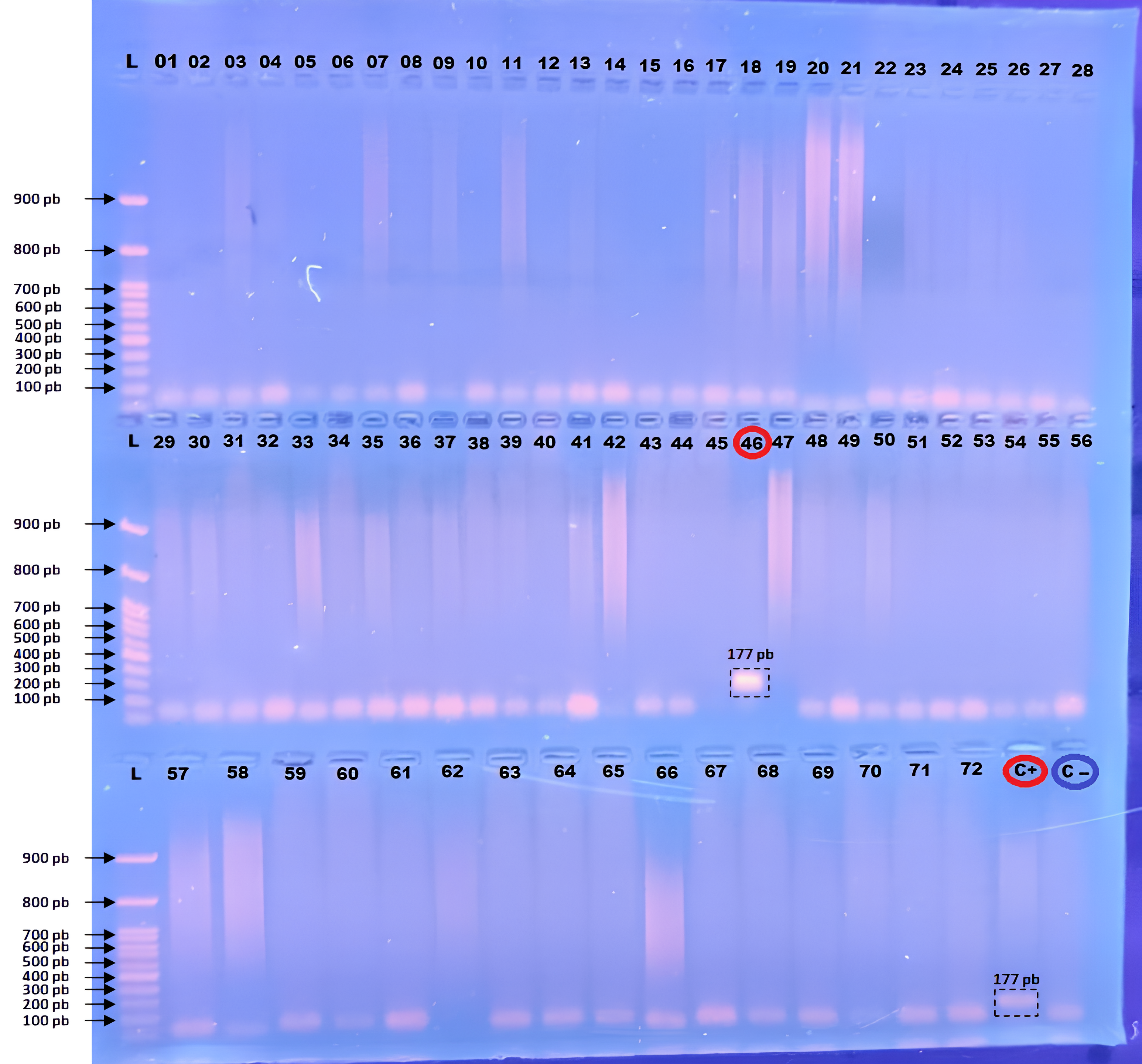

Supplement: Supplementary file 1 [file pathogens-15-00273-s001.zip › Figure S1.tif]
